# Novel *NR5A1* Missense Mutation in Premature Ovarian Failure: Detection in Han Chinese Indicates Causation in Different Ethnic Groups

**DOI:** 10.1371/journal.pone.0074759

**Published:** 2013-09-20

**Authors:** Xue Jiao, Yingying Qin, Guangyu Li, Shidou Zhao, Li You, Jinlong Ma, Joe Leigh Simpson, Zi-Jiang Chen

**Affiliations:** 1 Center for Reproductive Medicine, Provincial Hospital Affiliated to Shandong University, National Research Center for Assisted Reproductive Technology and Reproductive Genetics, The Key laboratory for Reproductive Endocrinology of Ministry of Education, Shandong Provincial Key Laboratory of Reproductive Medicine, Jinan, China; 2 Research and Global Programs March of Dimes Foundation, White Plains, New York, United States of America; 3 Human and Molecular Genetics, Obstetrics and Gynecology, Herbert Wertheim College of Medicine, Florida International University, Miami, Florida, United States of America; 4 Center for Reproductive Medicine, Renji Hospital, Shanghai Jiao Tong University School of Medicine, Shanghai Key Laboratory for Assisted Reproduction and Reproductive Genetics, Shanghai, China; Institute of Zoology, Chinese Academy of Sciences, China

## Abstract

**Background:**

The etiology of most premature ovarian failure (POF) cases is usually elusive. Although genetic causes clearly exist and a likely susceptible region of 8q22.3 has been discovered, no predominant explanation exists for POF. More recently, evidences have indicated that mutations in *NR5A1* gene could be causative for POF. We therefore screened for mutations in the *NR5A1* gene in a large cohort of Chinese women with non-syndromic POF.

**Methods:**

Mutation screening of *NR5A1* gene was performed in 400 Han Chinese women with well-defined 46,XX idiopathic non-syndromic POF and 400 controls. Subsequently, functional characterization of the novel mutation identified was evaluated in vitro.

**Results:**

A novel heterozygous missense mutation [c.13T>G (p.Tyr5Asp)] in *NR5A1* was identified in 1 of 384 patients (0.26%). This mutation impaired transcriptional activation on *Amh*, *Inhibin-a*, *Cyp11a1* and *Cyp19a1* gene, as shown by transactivation assays. However, no dominant negative effect was observed, nor was there impact on protein expression and nuclear localization.

**Conclusions:**

This novel mutation p.Tyr5Asp, in a novel non-domain region, is presumed to result in haploinsufficiency. Irrespectively, perturbation in *NR5A1* is not a common explanation for POF in Chinese.

## Introduction

Premature ovarian failure (POF), also termed primary ovarian insufficiency (POI), refers to cessation of normal ovarian function before the age of 40 years. Approximately 1% of the population has POF prior to age 40 and only 0.1% or less before age 30 [1], [2]. The etiology of POF is highly heterogeneous. Chromosomal abnormalities account for 12% of cases [3], and the familial aggregation often associated with POF indicates a genetic contribution. Causative mutations in several genes (e.g., *NOBOX*, *FIGLA*, *BMP15* and *GDF9*) have been identified in non-syndromic POF [4]–[8], but none of these genes are perturbed in more than a small minority of POF cases, in a given ethnic group. More recently, our genome-wide association study (GWAS) of POF discovered a significantly susceptible region of 8q22.3; however, it was concluded as an important yet undefined long distance regulatory region affecting oogenesis [9]. Therefore, the underlying explanation for POF remains largely unknown (idiopathic).

Nuclear receptor subfamily 5, group A, member 1 (*NR5A1*, MIM#184757), at human chromosome 9q33, encodes steroidogenic factor 1 (SF1), a nuclear receptor involved in adrenal and gonadal development, steroidogenesis, and reproduction [10]. SF1 serves as a master transcriptional regulator of multiple genes, including *STAR*, *CYP11A1*, *CYP19A1*, *AMH*, *INHA*, and *LHB* [11]–[15]. Human SF1 protein is mainly expressed in ovarian somatic cells of growing follicles and corpus lutea [16]. Granulosa cell-specific *Nr5a1* knockout mice exhibit infertility, hypoplastic ovaries with decreased follicles and absence of corpora lutea [17]. These findings indicate a critical role of *NR5A1* in ovarian development and function.

In humans, *NR5A1* mutations are associated with a wide range of phenotypes. Heterozygous, or rarely homozygous, variants were described in 46,XY disorders of sex development (DSD), with (rare) or without adrenal insufficiency, and milder phenotypes–hypospadias, cryptorchidism, anorchidia, and male infertility [18]. A pathogenic role of *NR5A1* in 46,XX POF has also been observed. Lourenco et al. [19] reported 19 different mutations in 4 families in which affected members had 46,XY DSD or 46,XX POF, and 2 out of 25 (8.0%) sporadic POF cases. In contrast, subsequent cohort studies demonstrated only a minor contribution of *NR5A1* to POF in several different populations [20]–[23]. Janse et al. found 1.4% in New Zealander (mutation carriers from Asian, Caucasian and Mediterranean); Lakhal et al. found no mutations in Tunisian but Philibert et al. found 3.8% in Tunisian. And more recently Voican et al. reported the mutation rate to be 1.6% in French.

Given these discrepant results, we screened the *NR5A1* gene in a large cohort of Chinese women with POF. Our cohort is the largest collected in any ethnic group and distinct in being exclusively Han Chinese. We found one novel heterozygous missense mutation, which was shown to impact functional significance with impaired transactivation activity.

## Methods

### Patients

A total of 400 unrelated Han Chinese women with POF were recruited from Center for Reproductive Medicine, Shandong Provincial Hospital Affiliated to Shandong University between April 2003 and June 2012. Inclusion criteria included primary amenorrhea (PA) or secondary amenorrhea (SA) for at least 6 months prior to the age of 40 years and two measures of serum FSH >40 IU/L obtained at least 1 month apart. In this cohort, 35 presented with PA and 365 with SA. A positive family history was considered if another first- or second-degree female family member had POF or early menopause (menopause before 45 years old). Patients with karyotypic abnormalities, previous chemo/radio-therapy, ovarian surgery or autoimmune diseases known to induce POF were excluded. Women with accompanying somatic anomalies were sought and excluded, particularly any reported as associated with syndromic POF (e.g., blepharophimosis-ptosis-epicanthus inversus syndrome, neurosensory deafness and cerebellar ataxia). Four hundred Han Chinese females with regular menses and normal hormone level were enrolled as controls.

### Ethics Statement

The study was approved by the Institutional Review Board of Reproductive Medicine of Shandong University. Written informed consent was obtained from each subject.

### Mutation Screening of *NR5A1* Gene

Genomic DNA was extracted from peripheral blood according to standard protocols. The entire coding sequence (exon 2–7) and intron-exon boundaries of *NR5A1* gene (NM_004959.4) were PCR amplified and directly sequenced (ABI 3730×l DNA Analyzer, Applied Biosystems, Foster City, CA). Novel variants identified were confirmed by bidirectional sequencing from another two independent PCR products. Nomenclature of variants identified was established according to Human Genome Variation Society (HGVS, www.hgvs.org/mutnomen). Primers and PCR conditions are available upon request.

### Plasmids Construction

SF1 expression vector containing p.Y5D mutation was generated by site-directed mutagenesis (QuikChange Lightning Site-Directed Mutagenesis Kit; Stratagene, LaJolla, CA) with wild-type (WT) mouse Sf1 expression vector as a template (a generous gift from Prof. Jameson). The luciferase reporter vectors containing SF1-responsive minimal promoters of murine Amh, murine Inhibin-a, murine Cyp11a1, and rat Cyp19a1 in pGL3-basic (Promega, Madison, WI, USA) were constructed. For expression and cellular localization, mouse *Nr5a1* cDNA was amplified and cloned in-frame into pEGFP-C3 expression vector (Clontech, Mountain View, CA), resulting in a fusion protein with a GFP tag at its N-terminus (pEGFP-C3-Sf1). The p.Y5D mutation was introduced as described above. All constructs were validated by sequencing. Primers used are detailed in Table S1.

### Transactivation Activity Assays

Transactivation activity assays were performed in 48-well plates using Lipofectamine ™ 2000 reagent (Invitrogen, Carlsbad, CA) and a Dual-Luciferase® reporter assay system (Promega, Madison, WI, USA). Human embryonic kidney HEK293T cells were transiently co-transfected by empty (Mock), wild-type (WT), or mutant (MT) Sf1 expression vectors with reporter constructs described above. To further assess the possible dominant negative effect, the WT vector with either empty vector or the mutant (1∶1) was transfected with each reporter. In addition, increasing mutant vectors (0, 10, 20, 30, 40 ng) in the absence or presence of constant WT (10 ng) and Amh-pGL3 reporter (540 ng) were co-transfected. Total transfected plasmids were adjusted with empty vectors. *Renilla* reporter pRL-TK (Promega) was used as an internal control. Cells were lysed 24 hours later, and luciferase activity was measured with a luminometer reader (Enspire, PerkinElmer). Results were normalized against *Renilla* luciferase activity.

### Expression and Nuclear Localization

HEK293T cells were transfected either with empty vector (Mock), WT or with mutant pEGFP-C3-Sf1 expression vector. Twenty-four hours after transfection, nuclear counterstaining was performed with Hoechst 33342 (Beyotime Institute of Biotechnology, Jiangsu, China), and then visualized under a fluorescence microscope (Olympus, Tokyo, Japan).

### Statistics

Results in transactivation activity assays were expressed as percentage of WT activity (100%) and represent the Mean±S.D. of 3 independent experiments, each performed in triplicate. Statistical significance was examined using Student’s t-test. Chi-square test was used for comparison of allele frequencies. A P value <0.05 was considered statistically significant.

## Results

### A Novel Missense Mutation Identified within Gene *NR5A1*

**Figure 1:**
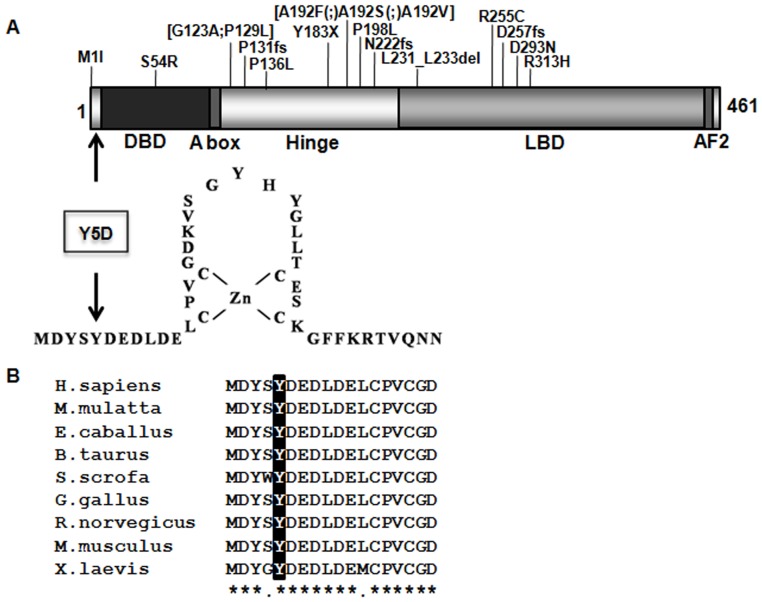
Mutations in *NR5A1* gene associated with POF. (A). Schematic presentation of the distribution of *NR5A1* mutations associated with POF. DBD: DNA-binding domain, LBD: ligand binding domain, AF2: activation function domain 2. (B). Sequence alignment of SF1 among orthologs with tyrosine residue highlighted.

The clinical characteristics of the 400 patients studied are summarized in Table 1. Direct sequencing of *NR5A1* gene was successful in 384 POF patients. A novel heterozygous missense mutation c.13T>G (p.Tyr5Asp, p.Y5D) in exon 2 was identified in one individual. This variant was absent in dbSNP database or 400 Chinese control women. The mutated tyrosine, located seven residues upstream of the first zinc finger DNA-binding domain (DBD), was highly conserved among SF1 orthologs (Fig. 1).

**Table 1:** Clinical characteristics of 400 Chinese women with POF.

Characteristics	Mean±S.D./N (%)
**Age at diagnosis (yr)**	29.82±4.95
**Age at menarche (yr)** a	14.85±2.20
**Age of amenorrhea (yr)** a	24.39±5.86
**FSH (IU/L)**	76.09±29.82
**E2 (pmol/L)**	25.38±42.42
**Family history**	41(10.3%)
**Parental consanguinity**	4(1.0%)

a Refer to patients with secondary amenorrhea.

The single p.Y5D carrier developed spontaneous menarche at the age of 19, but experienced irregular menses and subsequent secondary amenorrhea 1 year later. Transvaginal ultrasonography showed hypoplastic uterus and non-detectable ovaries. FSH was significantly elevated (78.3 IU/L), while estradiol was undetectable. No other family member had POF or a form of 46,XY DSD.

Two known single nucleotide polymorphisms (SNPs), rs1110061 and rs2297605, were also identified. However, the allele frequencies of these SNPs in our POF cohort were not statistically different from those in the Asian population (P>0.05) (Table S2).

### Impaired Transactivation Activity of the p.Y5D Mutant

**Figure 2:**
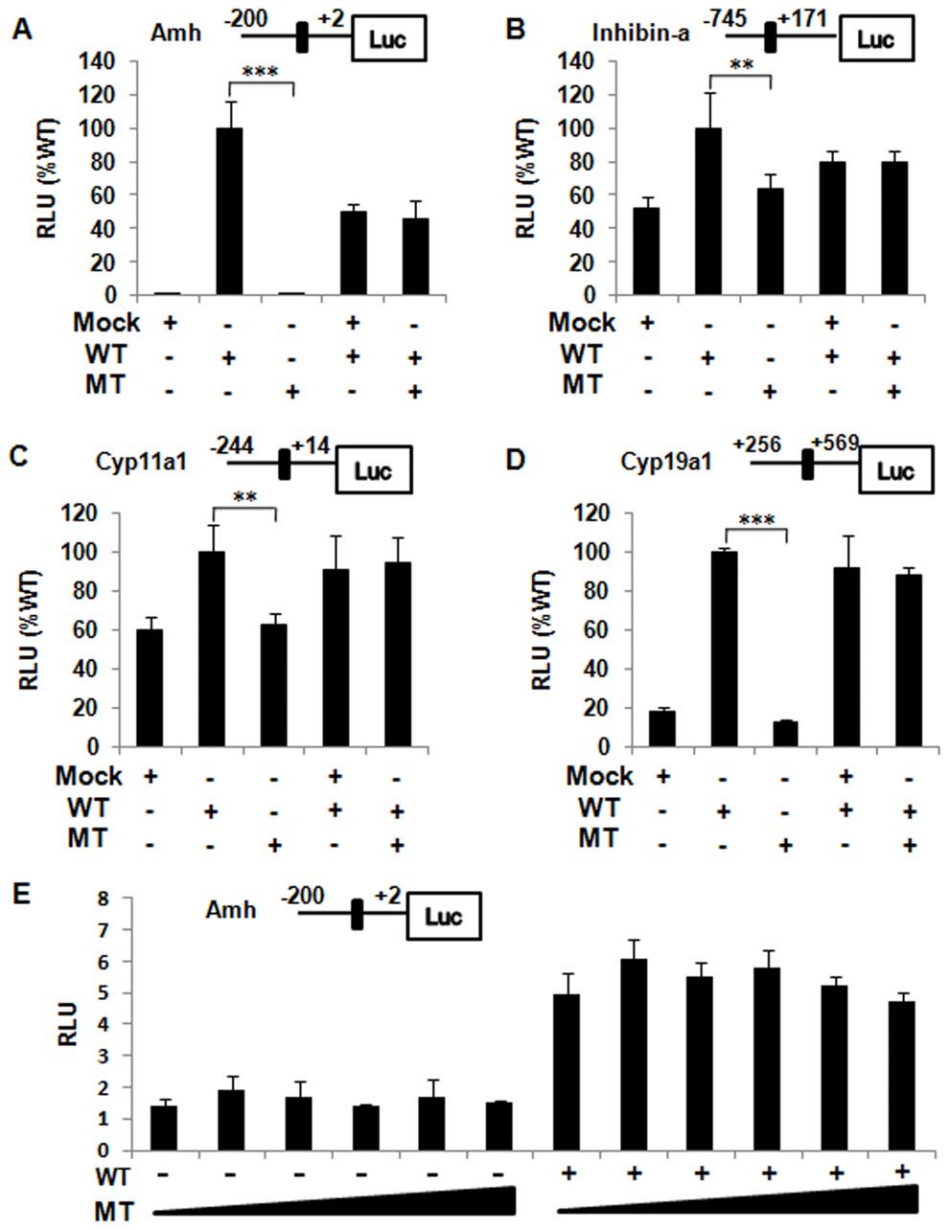
Transactivation activity assay. Co-transfection of empty (Mock), wild-type (WT), or mutant (MT) Sf1 expression vectors and a *Amh* (A), *Inhibin-a* (B), *Cyp11a1* (C) or *Cyp19a1* (D) promoter reporter were performed in HEK293T cells. Results are expressed as a percentage of WT activity (RLU WT%). The potential dominant negative effect of the p.Y5D mutant was assessed by co-transfecting WT expression vector with empty or MT vector (1∶1) (A, B, C, D) and increasing MT (0, 10, 20, 30, 40 ng) with 10 ng empty (−) or 10 ng WT (+) vector (Amh only) (E) in HEK293T cells. ** P<0.01, *** P<0.001. RLU, relative light units.

Transactivation activity assay showed that the p.Y5D mutation impaired the transactivation activity on different SF1 responsive promoter reporters, including Amh, Inhibin-a, Cyp11a1 and Cyp19a1 (all P<0.01) (Fig. 2A, B, C, D). Because of the heterozygous status of the patient, the dominant negative effect of the mutant over WT protein was assessed. Co-transfection of mutant with WT did not interfere with the transactivation of WT protein either when transfected in a 1∶1 ratio (Fig. 2A, B, C, D), or when increasing mutants transfected with a fixed WT even in ratio 5∶1(Amh only) (Fig. 2E).

### No Impact of the p.Y5D Mutation on Protein Expression and Nuclear Localization

**Figure 3:**
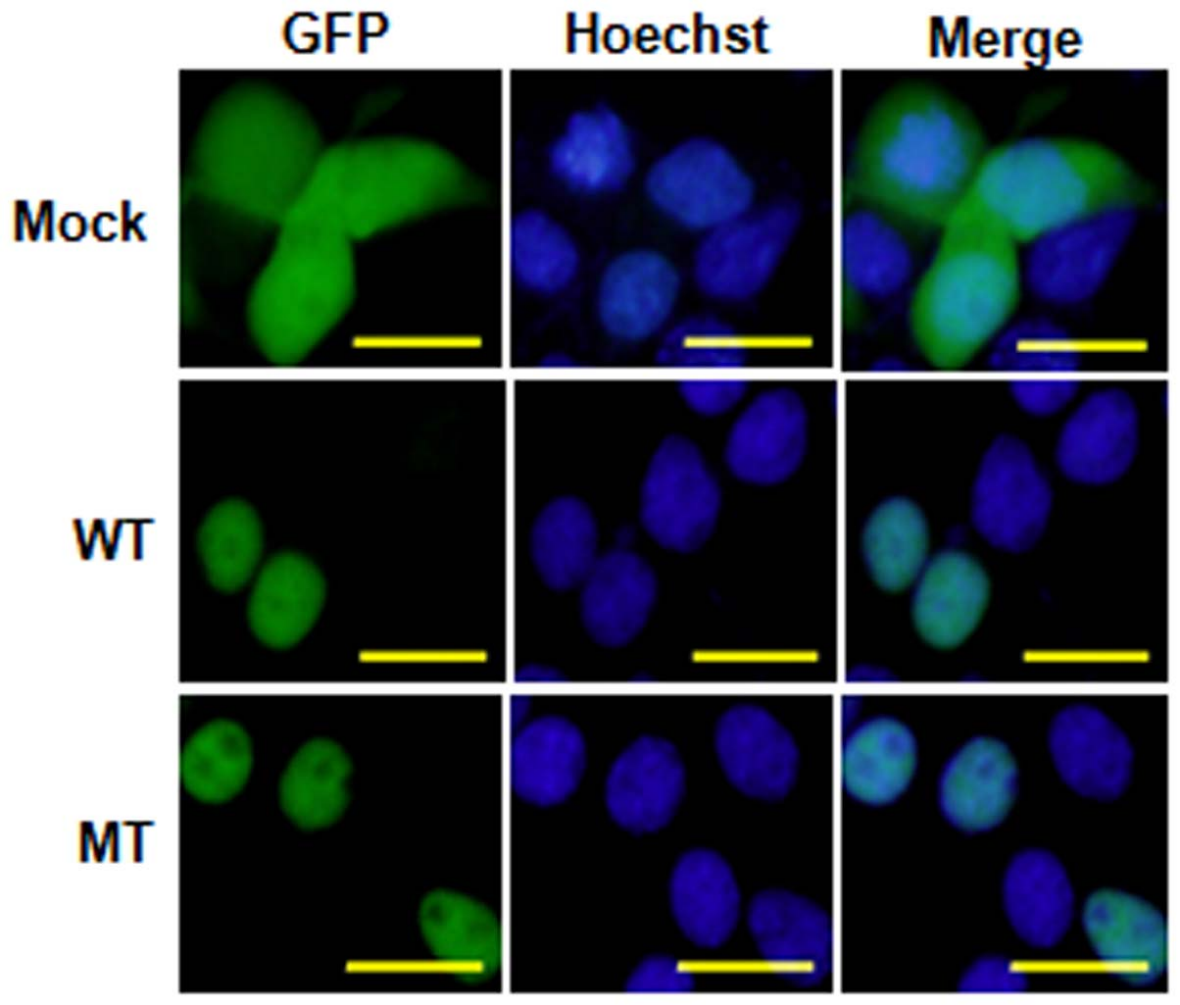
Expression and nuclear localization of p.Y5D mutant. HEK293T cells were transiently transfected with empty vector (Mock), wild-type (WT) or mutant (MT) pEGFP-C3-Sf1 expression vector. The nuclei were counterstained with Hoechst33342 (blue). Scale bars = 10 um.

To assess the impact of the p.Y5D mutation on protein expression and nuclear localization, we generated GFP-tagged Sf1 WT and mutant expression vectors and transfected them into HEK293T cells. The WT showed exclusively strong nuclear localization with relative nucleolar exclusion. A similar pattern was seen for the mutant (Fig. 3).

## Discussion

In our large cohort of Chinese patients known to have well-defined 46,XX non-syndromic POF, a novel heterozygous missense mutation was identified (1/384, 0.26%). The c.13T>G transition located in exon 2, outside any classic domains of SF1, resulted in a p.Tyr5Asp mutation. The highly conserved tyrosine residue was located adjacent to the first zinc finger of DBD, a region that contributed to specific recognition and interaction with promoter responsive elements in target genes. In vitro transactivation assays using Amh, Inhibin-a, Cyp11a1 and Cyp19a1 promoters showed impaired transactivation activity of the p.Y5D mutant, and thus confirmed its deleterious effect. However, a dominant negative effect was not observed (Fig. 2), consistent with previous reports [22], [24], [25].

The two SNP, rs1110061 and rs2297605, detected in our study had drawn attention of others. Rs1110061 (Gly146Ala) was reported to be associated with POF in recent cohorts of Tunisian women [21], [22]. Nevertheless, previous functional assays showed inconsistent results, that is, Gly146Ala resulted in either a decrease of 20–35% or no impact on the transactivation activity on target gene promoters [22], [25], [26]. Still, given that no significant difference existed in allele frequency between our POF cohort and the general Asian population, we considered rs1110061 to be only a polymorphism not conferring susceptibility to POF.

POF is genetically heterogeneous probably attributed to the complex genetic networks modulating human folliculogenesis and oogenesis. Ovarian phenotypes of *Nr5a1* null mice made it an excellent candidate gene for POF in human. *NR5A1/SF1* regulates the transcription of *CYP11A1*, *CYP19A1*, *AMH* and *INH-A* genes involved in steroidogenesis, ovarian development and function. Dysregulation of any protein encoded by these genes could lead to ovarian dysfunction. Disruption of *CYP11A1* (P450 Side Chain Cleavage, P450scc) or *CYP19A1* (Aromatase) could contribute to steroid hormones deficiency, estrogen in particular, and subsequent abnormal folliculogenesis. Firm evidence indicated an inhibitory role of *AMH* in initiation of primordial follicle growth and FSH responsiveness of small growing follicles [27], [28]. As an important negative regulator of pituitary FSH and paracrine factor in folliculogenesis, *INHIBIN-A* is also a downstream target of *NR5A1* [29]. It is plausible that a decline in AMH and inhibin levels would result in increased recruitment of primordial follicles, raised FSH or FSH responsiveness of small growing follicles, increased FSH-dependent cyclic recruitment, and hence, premature exhaustion of ovarian reserve occurred ultimately [27], [29]. However, we cannot exclude the possibility that other regulative pathways, even other genes, and/or environmental factors might participate in ovarian pathogenesis.

There have been five POF cohorts of different ethnicities in which *NR5A1* mutations were sought (Table 2). As discussed, the first identified two causative mutations among 25 sporadic POF cases (8.0%) [19]. In a large Dutch cohort of 356 POF cases, Janse et al. [20] later found five non-conservative mutations (1.4%) but nonetheless concluded that *NR5A1* made only a minor contribution to the pathogenesis for POF. Two Tunisian population were also studied, one finding no novel perturbations in 55 cases, the other finding 1 out of 26 cases (3.8%) [21], [22]. More recently, Voican et al. [23] reported three novel missense mutations in 180 French cases (1.6%). The present study revealed a much lower frequency of *NR5A1* mutations in Chinese (0.26%). Discrepant incidence might indicate differences in predisposing regulating pathways that lead to POF in ethnically diverse populations. In fact, similar inconsistency has been observed in other candidate genes for POF, extremely in FSHR, a frequent explanation of POF in Finland but rare elsewhere [30].

**Table 2:** Molecular and phenotypic features of 46,XX sporadic premature ovarian failure (POF) cases with *NR5A1* mutations.

Cases	Mutation rate (%)	Sequence variation^a^	Amino acid variation^a^	Location	Functional effect	Dominant negative	Age at diagnosis (yrs)	Ethnicity	Ref.
**PA(N = 35) SA(N = 365)**	1 (0.3)	c.13T>G	p.Y5D		Impaired transactivation on Amh, Inhibin-a,Cyp11a1 and Cyp19a1 promoters	No	20	Chinese	Current
**PA(N = 37) SA(N = 143)**	3 (1.6)	c.162C>A	p.S54R	DBD	No effect on DNA-binding capacity and transcriptional activity	No	20	Portugal	[23]
		c.593C>T	p.P198L	Hinge	No effect on DNA-binding capacity and transcriptional activity	No	33	North African	
		c.[368G>C; 386C>T]	p.[G123A; P129L]	Hinge	No effect on DNA-binding capacity and transcriptional activity	No	25	North African	
**PA(N = 26)**	1 (3.8)	c.763C>T	p.R255C	LBD	Sharp decrease in transactivation onCyp11a1 and Amh promoters	No	25	Tunisian	[22]
**PA(N = 25) SA(N = 30)**	0							Tunisian	[21]
**N = 2**	1	c.704C>T	p.P235L	LBD	80% of WT transcriptional activity onCYP17A1 and CYP11A1 promoters	No	15 (increased LH&FSH, undetectable E2&AMH, prepubertal uterus, nondetectable ovaries)	Swiss	[25]
**SA(N = 356)**	5 (1.4)	c.407C>T	p.P136L	Hinge	Not tested	Not tested		Asian, Caucasian	[20]
		c.[574G>T (;)575C>T]	p.[A192F(;)A192S(;) A192V]	Hinge	Not tested	Not tested		Mediterranean	
		c.593C>T	p.P198L	Hinge	Not tested	Not tested		Caucasian	
		c.938G>A	p.R313H	LBD	Not tested	Not tested		Caucasian	
**N = 25**	2 (8.0)	c.[368G>C; 386C>T]	p.[G123A; P129L]	Hinge	Severe loss of activation on CYP11A1and CYP19A1 promoters (P129L)No effect on transcriptional activity (G123A)	Not tested	12.5 (no development of breasts orpubic hair, fibrous ovary withoutfollicles, high FSH = 76 U/L)	Senegalese	[19]
		c.691_699del	p.L231_L233del	LBD	Severe loss of activation on CYP11A1and CYP19A1 promoters	Not tested	4 month (hypertrophic clitoris, high FSH = 44 U/L)	Italian	

a The table only refers to novel non-synonymous mutations and all mutations are heterozygous.

PA: primary amenorrhea; SA: secondary amenorrhea; DBD: DNA binding domain; LBD: ligand binding domain.

To date, 18 distinct variants have been reported in all patients with familial and sporadic POF (Table 2, 3, and Fig. 1) [19]–[25], [31], [32]. Mutations could be de novo or familial. All reported mutations were heterozygous, except p.Asp293Asn homozygosity in two Brazilian sibs [19]. Most mutations fell within the hinge region and ligand binding domain (LBD). Two were located outside any classic domains; these being a start codon mutation (p.Met1Ile) detected by Lourenco et al. [19] and the p.Tyr5Asp mutation in the present study. Both mutations affected highly-conserved residues. These findings underscore the likelihood of regulatory effects involving the non-domain region on gene function. Several variants resulted in impaired transcriptional activation, but without dominant negative effects. This suggests that haploinsufficiency alone may be sufficient to cause POF. Of note, phenotypic variability was remarkable between heterozygotes. Different individuals, even within the same family, having the same variant, presented with either premature ovarian failure, early menopause, diminished ovarian reserve (DOR), or normal ovarian function [31], [32]. In familial cases with 46,XY DSD and 46,XX POF, a heterozygous *NR5A1* mutation could be inherited from a 46,XX mother either with POF or apparently normal ovarian function, to the 46,XY DSD offspring in a sex-limited dominant manner, which was kind of similar to X-linked inheritance [18], [19], [31], [32] (Table 3). This information would be valuable in counseling even for sporadic cases with POF, because women with a heterozygous *NR5A1* mutation are at potential risk of passing it on to her 46,XY offspring.

**Table 3:** Molecular and phenotypic features of premature ovarian failure (POF) cases in 46,XY DSD families with *NR5A1* mutations.

Proband	Other affected family members	Sequence variation^a^	Amino acid variation^a^	Location	Functional effect	Dominant negative	Ethnicity	Ref.
**46,XY complete** **gonadal dysgenesis**	Mother: SA at 35 yrs	c.666delC	p.N222fs	Hinge	Severe loss of activation on CYP11A1and CYP19A1 promoters	Not tested	European	[19]
**46,XY DSD**	Sister: PA at 19 yrs	c.877G>A	p.D293N	LBD	Partially activate CYP11A1 and CYP19A1 promoters	Not tested	Brazilian	[19]
**46,XY partial gonadal dysgenesis**	Sister: SA at 16 yrs	c.3G>A	p.M1I		Not tested	Not tested	French	[19]
**46,XY DSD**	Mother: SA at 29 yrs	c.390delG	p.P131fs	Hinge	Not tested	Not tested	French	[19]
**46,XY DSD**	Mother: SA at 32 yrs	Unknown	p.Y183X	Hinge	Not tested	Not tested	Argentine	[31]
	Sister: regular menses, high FSH>40 U/L	Unknown	p.Y183X	Hinge	Not tested	Not tested	Argentine	
**46,XY DSD**	Mother: regular menses, high FSH>40 U/L	c.938G>A	p.R313H	LBD	Not tested	Not tested	Argentine	[32]
	Mother’s sister: regular menses, highFSH = 25.1 U/L	c.938G>A	p.R313H	LBD	Not tested	Not tested	Argentine	
**46,XY DSD**	Mother: SA at 38 yrs	c.768delC	p.D257fs	LBD	Decrease in transactivation on CYP11A1and CYP19A1 promoters	No	Japanese	[24]
	Mother’s mother: menopause at 38 yrs	c.768delC	p.D257fs	LBD	Decrease in transactivation on CYP11A1and CYP19A1 promoters	No	Japanese	

a All mutations are heterozygous except for c.877G>A (p.D293N).

46, XY DSD: 46, XY disorder of sex development; PA: primary amenorrhea; SA: secondary amenorrhea; LBD: ligand binding domain.

In summary, we identified a single novel heterozygous *NR5A1* mutation in a cohort of Chinese patients with 46,XX non-syndromic POF. This mutation, located in non-domain region, was transcriptionally impaired without a dominant negative effect. Although *NR5A1* perturbations appear to be a less common explanation for POF, our identifying a causative mutation, in an ethnic group (Han Chinese) different from that of other reports of mutations, strengthens the case for *NR5A1* playing a crucial role in ovarian development and function in some women. Comprehensive researches on signaling pathways associated to *NR5A1* in ovary are warranted to demonstrate its plausible causative roles in human POF.

## Supporting Information

Table S1 **Primers used in plasmids construction.**
(DOCX)

Table S2 **Known single nucleotide polymorphisms (SNPs) identified in our POF cohort.**
(DOCX)
