# Supplementary material for: Novel NR5A1 Missense Mutation in Premature Ovarian Failure: Detection in Han Chinese Indicates Causation in Different Ethnic Groups
Source: PLoS One. 2013 Sep 20;8(9):e74759. doi: 10.1371/journal.pone.0074759 (PMC3779243; doi:10.1371/journal.pone.0074759)
Supplement: Table S1 — Primers used in plasmids construction. (DOCX) [file pone.0074759.s001.docx]

**Table S1.** Primers used in plasmids construction.

| **Primer** | | **Sequence** | |
| --- | --- | --- | --- |
| Sf1-Y5D-F | GGCATGGACTATTCGGACGACGAGGACCTGG | |  |
| Sf1-Y5D-R | CCAGGTCCTCGTCGTCCGAATAGTCCATGCC | |  |
| pEGFP-C3-Sf1-F | CCCAAGCTTATGGACTATTCGTACGACGAG | |  |
| pEGFP-C3-Sf1-R | CGCGGATCCTCAAGTCTGCTTGGCCTGCA | |  |
| Amh-F | GAGCTCCTCAGGCCTCTGCAGTTATGG | |  |
| Amh -R | AAGCTTATGGTGGTACAGCAAGGTCC | |  |
| Inhibin-a-F | CTCTTTACCCTGGACCCT | |  |
| Inhibin-a-R | AGTTCACAACGGGACTAC | |  |
| Cyp11a1-F | CATAGGGTGGACACTGAGGTTAG | |  |
| Cyp11a1-R | CACGACTGCCACTTCCTGCT | |  |
| Cyp19a1-F | GCCTTTACCTGCTCTTGATTTG | |  |
| Cyp19a1-R | GGAAGAAAGCAGTAGTGATGTTAGC | |  |
