# Supplementary material for: Novel NR5A1 Missense Mutation in Premature Ovarian Failure: Detection in Han Chinese Indicates Causation in Different Ethnic Groups
Source: PLoS One. 2013 Sep 20;8(9):e74759. doi: 10.1371/journal.pone.0074759 (PMC3779243; doi:10.1371/journal.pone.0074759)
Supplement: Table S2 — Known single nucleotide polymorphisms (SNPs) identified in our POF cohort. (DOCX) [file pone.0074759.s002.docx]

**Table S2.** Known single nucleotide polymorphisms (SNPs) identified in our POF cohort.

|  |  |  | **Genotype frequency (%)** | | **Allele frequency (%)** | |  |
| --- | --- | --- | --- | --- | --- | --- | --- |
| **Variation** | **Location** | **dbSNP ID** | **POF** | **Asian population^a^** | **POF** | **Asian population^a^** | **P value^b^** |
| c.437 G>C | Exon4 | rs1110061 | GG (47.9) | NA | G (71.4) | G (72.5) | >0.05 |
| p.Gly146Ala |  |  | GC (46.9) | NA | C (28.6) | C (27.5) |  |
|  |  |  | CC (5.2) | NA |  |  |  |
| c.871-20C>T | Intron4 | rs2297605 | TT(31) | NA | T (56.8) | T (57.5) | >0.05 |
|  |  |  | TC(51.6) | NA | C (43.2) | C (42.5) |  |
|  |  |  | CC(17.4) | NA |  |  |  |

NA: Not available.

^a^Data from http://www.ncbi.nlm.nih.gov/projects/SNP/

^b^Comparison of the allele frequency between patients with POF and Asian population.
